# Effects of Guanidine Acetic Acid on the Growth and Slaughter Performance, Meat Quality, Antioxidant Capacity, and Cecal Microbiota of Broiler Chickens

**DOI:** 10.3390/vetsci11110550

**Published:** 2024-11-08

**Authors:** Xuedan Li, Zhimin Chen, Jiantao Li

**Affiliations:** 1 School of Animal Science and Medicine, Shenyang Agricultural University, Shenyang 110866, China; 18241807285@163.com; 2 Key Laboratory for Feed Biotechnology of the Ministry of Agriculture and Rural Affairs, Institute of Feed Research, Chinese Academy of Agriculture Sciences, Beijing 100081, China

**Keywords:** arbor acres broiler chickens, antioxidant capacity, growth performance, guanidine acetic acid, meat quality, slaughter performance

## Abstract

The objective of this research was to assess the impact of guanidine acetic acid (GAA) on the growth performance, slaughter outcomes, meat quality, antioxidant capacity, and cecal microbiota of broiler chickens. A total of 128 Arbor Acres broilers were randomly divided into two experimental groups. One group served as the control and was provided with a standard diet, whereas the group treated with GAA received a diet enhanced with 400 mg/kg of GAA. The duration of the experiment was 42 days. Measurements for growth performance, serum biochemical parameters, and antioxidant capacity were conducted both during and at the conclusion of the study, while assessments of slaughter performance and meat quality were carried out solely at the end. Notable differences were observed in terms of growth performance, blood biochemistry, and metabolic parameters between the control and GAA-treated groups (*p* < 0.05). Hence, these findings imply that dietary GAA supplementation can favorably affect growth, carcass quality, biochemical indicators, and antioxidant capacity in broiler chickens.

## Simple Summary

Guanidine Acetic Acid (GAA) is an innovative feed additive that has attracted considerable interest from scientists because of its distinctive physiological benefits and extensive potential for application. Serving as an essential precursor for the internal production of creatine in animals, GAA is vital for fostering muscle development, boosting energy metabolism, and aiding in protein synthesis. In this investigation, incorporating 400 mg/kg of GAA resulted in a notable enhancement in the growth performance of broiler chickens while lowering percentages of abdominal fat, muscle drip, and cooking losses. Additionally, GAA effectively modulates serum biochemical parameters in broiler chickens, improves antioxidant capabilities, and reduces levels of lipid peroxidation. Nonetheless, GAA did not have a significant impact on the microbial composition and diversity within this group of chickens. As a result, the findings from this study indicate that the incorporation of 400 mg/kg GAA can substantially enhance the growth and development of broiler chickens.

## 1. Introduction

The livestock and poultry sectors are encountering major challenges due to increasing apprehensions about antibiotic misuse, food safety, and animal welfare on a global scale [1,2]. Measures designed to mitigate antibiotic resistance have been introduced to lower antibiotic residues in meat products, safeguard human health, and uphold ecological equilibrium [3,4]. Nonetheless, these measures have resulted in complications related to disease prevention and management, as well as promoting growth in animal agriculture [5,6]. In response to these challenges, studies concentrating on finding and implementing safe and effective alternatives to antibiotics have been undertaken.

Guanidine acetic acid (GAA), a non-antibiotic growth promoter, has attracted considerable attention from researchers and stakeholders of livestock and poultry industries because of its unique biological functions and various potential applications [7]. GAA facilitates creatine synthesis, accelerates metabolism, and promotes the growth and maturation of livestock and poultry [8]. Moreover, GAA is involved in regulating the immune system, enhancing disease resistance in animals, and reducing the occurrence and spread of diseases [9]. Most importantly, as GAA is non-toxic, easily degradable, and does not leave any residues, it satisfies the requirements of the livestock industry for environment-friendly, safe, and efficient interventions [10]. Previous research indicates that guanidinoacetic acid (GAA) can markedly boost the growth and development, improve meat quality, and enhance the antioxidant properties in livestock, including pigs and lambs [11,12,13,14]. Furthermore, studies have noted that GAA contributes to better nutrient utilization and improves the meat quality in broiler chickens [8,15]. The addition of GAA to diets with low metabolic energy can lead to improved efficiency in energy utilization, subsequently enhancing the growth performance of broiler chickens [16]. Moreover, GAA has the capability to mitigate the reduced growth performance of broiler chickens caused by heat stress [17,18]. Nonetheless, variations exist in the reported impacts of different forms and quantities of GAA on the growth and development of broilers.

Consequently, this research aimed to assess the influence of GAA on various aspects such as growth, slaughter performance, meat quality, serum biochemical markers, antioxidant capacity, and cecal microbiota in broiler chickens. The results from this investigation provide a theoretical basis for implementing GAA in the production of broiler chickens.

## 2. Materials and Methods

### 2.1. Ethics Statement

This study was approved by the Animal Care and Use Committee of the School of Animal Science and Medicine, Shenyang Agricultural University (NO. 2023051001).

### 2.2. Animals and Diets

A total of 128 Arbor Acres (41.34 ± 1.21 g) broiler chickens were selected and randomly divided into 2 experimental groups, with 8 replicates in each group and 8 chickens in each replicate. The control group (CON) was fed with basic feed (Table 1) configured according to the nutrient requirements of poultry stipulated in NRC1994 and NY-T33-2004. Meanwhile, the treatment group (hereafter referred to as the GAA-treated group) was provided with a diet similar to that of the CON group but was supplemented with 400 mg/kg GAA. The experimental period lasted for 42 d. The entire experiment was performed using a three-layer, fully stepped cage-raising method. Prior to the experiment, the chicken coops were thoroughly cleaned, disinfected, and left empty. On the day when the chicks entered the coops, the temperature inside the chicken shed ranged from 32 °C to 33 °C. The temperature was decreased by 1 °C every 3 d; it was maintained between 20 °C and 21 °C after 34 d. The relative humidity in the chicken coops was maintained between 55% and 65% from days 1 to 21; it was maintained below 55% from days 22 to 42. From days 1 to 3, the chicken coops were exposed to light for 24 h. From days 23 to 30, the coops were exposed to light for 5 h; beyond day 30, the coops were exposed to light for 24 h. The chickens were fed 3 times a day (8:00, 14:00, and 20:00), and the tank was emptied for 1 h before each feeding. Nipple-style water dispensers were used for water supply. The feces of the chickens were regularly cleaned.

### 2.3. Growth Performance

The average daily gain (ADG) and average daily feed intake (ADFI) of the broiler chickens from days 1 to 21, 22 to 42, and 1 to 42 were measured; afterwards, the feed-to-gain (F/G) ratio was calculated. To correct for chick F/G ratio, the number of dead chicks was recorded and their body weights were measured daily.

### 2.4. Slaughter Performance

On the 42nd day of the feeding trial, following the standards specified in the “Terminology and Statistical Methods for Measurement of Poultry Production Performance” (NY/T823-2020) [19], a chicken of moderate weight was randomly chosen from each replicate to serve as the subject for the experiment. After fasting for 12 h, measurements were taken for the slaughter weight, semi-eviscerated weight, eviscerated carcass weight, weight of the pectoral muscle, weight of the leg muscle, and abdominal fat weight of the selected chickens. Following this, the dressing percentage (DP), semi-eviscerated weight rate (SEWR), eviscerated carcass weight rate (ECWR), pectoral muscle rate (PMR), leg muscle rate (LMR), and abdominal fat rate (AFR) were subsequently determined.

### 2.5. Meat Quality

On day 42 of the experiment, one healthy broiler chicken from each replicate, each with similar weights were selected and assessed in terms of the following meat properties: brightness (BRT), mature meat rate (MMR), shear force (SF), and drip loss (DL). To measure meat color, muscles of uniform size and thickness were selected, and a colorimeter was used to measure the L value (brightness) of the meat color index at three different positions; the average of three measurements was recorded. DL was measured by weighing a long strip of a meat sample and suspending it in a 550 mL dry plastic bottle. The bottle was sealed and then stored in a refrigerator at 4 °C. The weight of the muscle samples was measured at 24 h intervals; the final measurement was conducted at 48 h to calculate the DL rate. Cooked meat rate was determined by weighing a 30 g muscle sample, placing it in a self-sealing bag, and boiling it in a water bath at 80 °C for 30 min. The samples were weighed after they had cooled down. The cooked meat percentage was calculated as the ratio of muscle weight after cooking to that before cooking. SF was measured by taking a long muscle sample; after the muscle fat, tendons, and membrane had been removed, a tenderness meter was used to shear the sample six times, and the average value was recorded.

### 2.6. Serum Biochemical Indicators

On days 21 and 42 of this study, two broiler chickens that shared comparable weights were chosen from each replicate group for analysis. After a fasting period of 12 h, these chickens underwent blood collection through the wing vein. The collected blood samples were then subjected to centrifugation at a force of 3000× *g* and a temperature of 4 degrees Celsius for a duration of 15 min. Following this process, the separated serum samples were stored at a temperature of −80 degrees Celsius to preserve their integrity for later evaluation. The analysis included the quantification of key biochemical markers in the serum. Specifically, the levels of total protein (TP), triglyceride (TG), high-density lipoprotein (HDL), low-density lipoprotein (LDL), insulin (INS), chicken growth hormone (CGH), and the thyroid hormones T3 and T4 were determined using specialized reagent kits. In addition to these parameters, several serum antioxidant indicators were also evaluated. These included total antioxidant capacity (T-AOC), superoxide dismutase (SOD), malondialdehyde (MDA), and glutathione peroxidase (GSH-Px), all of which were measured utilizing reagent kits sourced from the Nanjing Jiancheng Biotechnology Research Institute located in Nanjing, China.

### 2.7. Cecal Microbiota

At 42d, three broiler chickens with comparable weights were selected for each treatment group to facilitate the collection of cecal digestates. The digestive material obtained from the cecum was immediately frozen in liquid nitrogen to preserve its integrity and subsequently stored at a temperature of −80 °C for future analysis. To isolate genomic DNA from the cecal digesta samples, the EZNA stool DNA kit provided by Omega Biotek (Norcross, GA, USA) was utilized. The concentration of the extracted DNA was determined using a NanoDrop 2000 spectrophotometer (Thermo Scientific, Waltham, MA, USA), while its quality was assessed through electrophoresis performed on 2% agarose gels. Following the DNA extraction and quality assessment, the V3–V4 region of the 16S rRNA gene was amplified. This amplification utilized genomic DNA extracted from the cecal digesta samples as a template, employing specific primers 338F (5′barcodeACTCCTACGGGAGGCAGCAG3′) and 806R (5′GGACTACHVGGGTWTCTAAT3′). To purify the resultant PCR products, the AxyPrep DNA Gel Extraction Kit from Axygen Biosciences (Union City, CA, USA) was employed. The purified amplicons were then pooled in equal molar ratios to prepare for sequencing, which was conducted using the Illumina MiSeq platform. To ensure the integrity of the sequence data, the raw Illumina fastq files underwent a quality-filtering process using Trimmomatic (version 3.29). Subsequently, operational taxonomic units (OTUs) were clustered at a threshold of 97% similarity, utilizing UPARSE (version 7.0), which allowed for effective categorization of the identified microbial communities.

### 2.8. Statistical Analysis

To check for normal distribution and variances’ homogeneity, Shapiro–Wilk and Levene’s tests were conducted on the data. The independent t-test was utilized for data analysis. Results are presented as the mean along with the standard error of the mean. A significance level of *p* < 0.05 was established. The analysis of broiler gut microbiota was carried out using the Majorbio Cloud Platform’s online services (https://www.majorbio.com).

## 3. Results

### 3.1. Growth Performance

Table 2 demonstrates that from days 22 to 42, ADG and ADFI significantly increased (*p* < 0.05), and the F/G ratio significantly decreased in the GAA-treated group (*p* < 0.05).

### 3.2. Slaughter Performance

Table 3 demonstrates that there was no significant difference in DP, SEWR, ECWR, PMR, and LMR (*p* > 0.05) between the CON and GAA-treated groups; however, AFR significantly decreased (*p* < 0.05) in the GAA-treated group.

### 3.3. Meat Quality

Table 4 demonstrates that compared with the CON group, SF and DL significantly decreased (*p* < 0.05) in the GAA-treated group.

### 3.4. Serum Biochemical Parameters

Table 5 illustrates that on day 21, the GAA-treated group displayed significantly increased serum levels of CGH, T3, and T4 in comparison to the CON group (*p* < 0.05); however, no significant difference was observed in the serum levels of TP, HDL, LDL, and INS between the CON group and the GAA-treated group. By day 42, measurements indicated that the serum concentrations of HDL, LDL, CGH, T3, and T4 in the GAA-treated group were significantly greater (*p* < 0.05) while no significant differences were noted in the serum levels of TP and INS when comparing the CON and GAA-treated groups.

### 3.5. Antioxidant Capacity

Table 6 illustrates the influence of guanidine acetic acid on the antioxidant capacity present in the serum of broiler chickens. In comparison to the CON group, there was a notable increase (*p* < 0.05) observed in GSH-Px activity of the GAA group.

### 3.6. Cecal Microbiota

**Figure 1:**
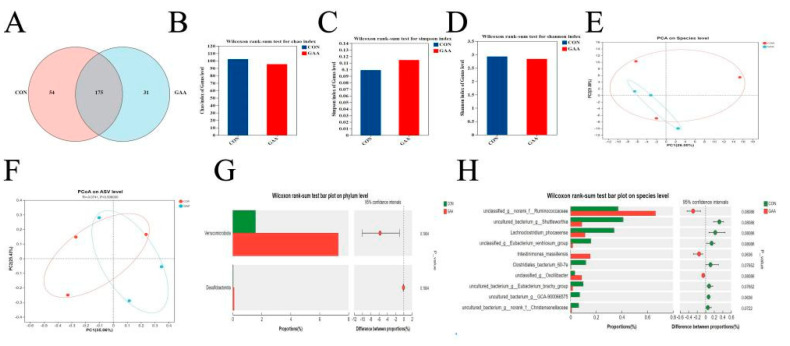
Microbial diversity analysis of cecum in broiler chickens. (**A**) Venn diagram; (**B**) Chao index; (**C**) Simpson index; (**D**) Shannon index; (**E**) PCA; (**F**) PCoA; (**G**) bar chart of relative abundance of species at the phylum level; (**H**) bar chart of relative abundance of species at the species level.

As shown in Figure 1, the CON group comprises 54 unique species, while the GAA group contains 31 unique species, resulting in a total of 175 unique species across both groups (Figure 1A). No significant differences (*p* > 0.05) were observed between the CON and GAA groups, as demonstrated by the Chao (Figure 1B), Simpson (Figure 1C), and Shannon (Figure 1D) indices, as well as PCA (Figure 1E), PCoA (Figure 1F), and the differences in species abundance at the phylum (Figure 1G) and genus (Figure 1H) levels.

## 4. Discussion

The growth performance of broilers encompasses their rate of growth, efficiency in feed conversion, and overall health condition throughout the rearing process; additionally, it serves as a crucial metric for assessing their production and economic gains [20,21]. Recently, there has been a quest for safer and more effective substitutes for antibiotics, prompted by the discovery of antibiotic residues in animal feed [22]. GAA has steadily emerged as a promising alternative to antibiotics due to its distinct biological properties [23]; for example, studies have shown that substituting antibiotics with GAA-enriched nutritional feed resulted in beneficial effects on broiler production outcomes [24,25]. Previous research indicates that diets supplemented with 600 mg/kg GAA led to a significant decrease in the feed conversion ratio of broilers [25]. Conversely, Li et al. [18] found no significant enhancement in the growth and development of broiler chickens when feed was augmented with 600 mg/kg GAA. This variation in findings could be linked to factors such as the age and breed of the test chickens, along with their differing responses to GAA supplementation. In the current study, incorporating 400 mg/kg GAA into the diet of the broiler chickens notably enhanced their average daily gain (ADG) and average daily feed intake (ADFI) on days 21 and 42, while concurrently reducing the feed-to-gain (F/G) ratio. GAA functions as a precursor to creatine, which is abundantly found in muscle and nerve tissues, and it serves as a primary energy source for muscle [26,27]. The inclusion of GAA in the diet could lead to an increased production of phosphocreatine in the body, thus facilitating a sustained energy supply to muscles [28]. This mechanism may account for the enhancement of growth performance in broiler chickens due to GAA.

Slaughter performance serves as a crucial metric for evaluating the meat production capabilities of livestock and poultry [29,30]. The significance of various sub-parameters related to slaughter performance can be outlined as follows: SEWR and ECWR serve as direct indicators of meat production efficiency in these animals [31]. Meanwhile, PMR and LMR provide insights into muscle development and the quality of the meat produced, while AFR reflects the level of fat accumulation in livestock and poultry [32]. Prior research indicated that a dosage of 600 mg/kg GAA does not notably impact the slaughter performance of broiler chickens [25]. In contrast, findings from this study demonstrate that the inclusion of 400 mg/kg GAA in the feed resulted in a significant decrease in the AFR of broiler chickens. GAA facilitates the oxidative metabolism of fatty acids, leading to greater utilization of body fat [33], which in turn diminishes fat storage and weight gain [34]. Additionally, GAA has the capacity to hinder fatty acid synthesis, lower blood lipid concentrations, and act as a preventive measure against obesity and metabolic disorders [35]. The dual regulatory influence of GAA on fat metabolism helps to decrease abdominal fat accumulation in broiler chickens.

SF and DL serve as critical indicators of meat quality [36]. More specifically, SF is primarily utilized to assess meat tenderness, whereas DL denotes variations in the water-holding capacity of muscle proteins stemming from water loss during meat processing and storage [37,38]. This study demonstrates that GAA considerably decreased the SF and DL levels in broiler muscles, suggesting its beneficial effects on both broiler growth and metabolism. Acting as a precursor to creatine, GAA facilitates the synthesis of creatine, which subsequently enhances the energy supply to the muscles. Consequently, GAA contributes to the improvement of meat texture and flavor, rendering it more tender. Furthermore, elevated creatine levels assist in enhancing the water-retention capacity of muscles, which leads to diminished DL [39,40]. Additionally, GAA may aid in boosting the protein content of muscles, improve their nutritional profile, and lessen fat accumulation.

Supplementation with GAA resulted in a notable rise in the serum concentrations of HDL, LDL, CGH, T3, and T4 in broiler chickens while concurrently lowering the TG levels. HDL and LDL serve as the primary lipoproteins that facilitate cholesterol transport in the bloodstream. HDL is responsible for transporting cholesterol from peripheral tissues back to the liver for metabolism and plays a significant role in minimizing cholesterol accumulation on vascular walls [41]. The peptide CGH, synthesized and released by the pituitary gland, is crucial for the comprehensive regulation of the growth processes in chickens [42]. Furthermore, T3 and T4 are key components of thyroid hormones, which exert essential effects on the growth, developmental processes, metabolic regulation, and nervous system function in broilers [43]. Consequently, GAA enhances the physiological well-being of broiler chickens by influencing lipid metabolism, thyroid functionality, and other relevant processes. Additionally, the serum levels of T-AOC and SOD activity in broilers treated with GAA showed a marked increase, while a significant decrease in MDA levels was observed. In a similar investigation, Zhao et al. [25] reported that GAA supplementation led to elevated T-AOC activity and reduced reactive oxygen species and MDA levels in broiler chickens. T-AOC serves as a vital measure of the antioxidant strength provided by antioxidants and their corresponding enzymes [44,45]. Among these, SOD is a crucial antioxidant enzyme that primarily functions to eliminate free radicals from the body, thereby lessening the damage inflicted by these radicals on cellular structures [46]. In addition, the level of MDA serves as an indicator of lipid peroxidation extent and cellular injury within the body [47]. By boosting the activity of antioxidant enzymes and conserving antioxidants, GAA enhances the animals’ antioxidant capacity. Therefore, GAA contributes to safeguarding broiler chickens against oxidative stress, supporting their overall health and sustaining normal physiological functions.

The cecum plays an essential role in the intestinal system of broiler chickens, acting as the main location for the colonization of gut microorganisms and the fermentation of these anaerobic microbes [48]. The microbial community within the cecum of broiler chickens is a complex and important assemblage that significantly contributes to nutrient absorption, immune protection, and overall intestinal wellbeing [49]. In our study, incorporating 400 mg/kg GAA into the feed did not lead to notable changes in the alpha and beta diversity of microorganisms present in the cecum of broiler chickens. Additionally, when analyzing the relative abundance bar charts at both the phylum and species levels, no significant differences were found between the control group and the GAA group. Supporting our results, Zhao et al. noted that including 0.06% GAA in the diet did not significantly alter the gut microbiota [25]. Likewise, Li et al. found that the addition of GAA had no discernible impact on alpha diversity in ruminants, implying that GAA does not affect the richness or distribution of rumen microbiota in lambs [12]. We propose that, although GAA is acknowledged as a precursor to creatine and aids its synthesis, it may not directly alter microbial diversity. Any effects of GAA on cecal microbiota in broiler chickens might primarily occur through changes in metabolites. Nevertheless, this proposition requires additional investigation.

## 5. Conclusions

In conclusion, the inclusion of 400 mg/kg GAA in the feed notably improved growth performance and lowered the AFR, muscle SF, and DL in broiler chickens. Furthermore, GAA influenced the serum biochemical parameters, boosted antioxidant capacity, and mitigated lipid peroxidation levels in these chickens. Nevertheless, there was no considerable impact of GAA on the microbial composition and diversity among the broiler chickens.

## Figures and Tables

**Table 1:** Ingredients and nutrient content of experimental diets (%, as-is basis).

Items	Contents	
	Days 1 to 21	Days 22 to 42
Corn	54.34	59.00
Corn gluten meal	4.60	5.60
Extruded full-fat soybean	3.40	1.70
43% Soybean meal	30.23	25.69
Soybean oil	2.80	4.00
Limestone	1.25	1.20
CaHPO_4_	2.10	1.90
NaCl	0.22	0.30
Premix ^(1)^	0.18	0.18
*L*-Lysine	0.37	0.18
*DL*-Methionine	0.19	0.05
*L*-Threonine	0.05	0.00
*L*-Tryptophan	0.01	0.00
Valine	0.06	0.00
50% Choline chloride	0.10	0.10
Phaytase	0.02	0.02
Compound enzyme	0.06	0.06
Antioxidant	0.02	0.02
Nutrients ^(2)^		
Digestive energy, MJ/kg	12.83	13.33
Crude protein	21.62	19.27
Calcium, %	1.06	0.98
Nonphytate phosphorus	0.46	0.42
Lysine, %	1.33	1.02
Methionine, %	0.54	0.40
Threonine, %	0.85	0.73
Methionine + Cysteine, %	0.95	0.86

^(1)^ The premix provided the following per kg of diets: 10,000 IU VA, 2000 IU VD3, 10 IU VE, 2.5 mg VK_3_, 1.8 mg VB_1_, 4.0 mg VB_2_, 50 mg VB_3_, 0.7 mg VB_12_, 0.12 mg biotin, 8 mg Cu, 10 mg nicotinic acid, 11 mg pantothenic acid, 1.0 mg folic acid, 80 mg Fe, 60 mg Mn, 80 mg Zn, 0.35 mg I, and 0.30 mg Se. ^(2)^ Digestive energy was the calculated value, while the rest were the measured values.

**Table 2:** Effect of guanidine acetic acid on the growth performance of broiler chickens.

Items ^(1)^	ADG, g	ADFI, g	F/G
Days 1 to 21			
CON	29.98	40.26	1.34
GAA	29.42	39.36	1.34
SEM	0.145	0.337	0.015
*p*-value	0.447	0.167	0.989
Days 22 to 42			
CON	87.25 ^b^	139.89 ^b^	1.61 ^a^
GAA	93.21 ^a^	143.68 ^a^	1.57 ^b^
SEM	0.785	1.012	0.009
*p*-value	0.012	0.044	0.007
Days 1 to 42			
CON	60.20 ^b^	92.45 ^b^	1.54 ^a^
GAA	62.22 ^a^	94.07 ^a^	1.46 ^b^
SEM	0.471	0.559	0.012
*p*-value	0.029	< 0.001	< 0.001

^a,b^ Means followed by different letters in a row indicate a significant difference (*p* < 0.05), whereas similar letters in a row indicate no significant difference (*p* > 0.05). ^(1)^ ADG, average daily gain; ADFI, average daily feed intake; F/G, feed-to-gain. CON group, basal diet in the control group; GAA group, supplemented with 400 mg/kg guanidine acetic acid based on the control group diet; SEM, standard error of the mean.

**Table 3:** Effect of guanidine acetic acid on the slaughter performance of broiler chickens.

Items ^(1)^	DP, %	SEWR, %	ECWR, %	PMR, %	LMR, %	AFR, %
CON	93.49	89.36	77.61	24.22	25.54	0.58 ^a^
GAA	92.82	87.30	76.87	23.87	25.13	0.53 ^b^
SEM	0.158	0.281	0.224	0.612	0.227	0.041
*p*-value	0.413	0.079	0.217	0.117	0.671	0.032

^a,b^ Means followed by different letters in a row indicate a significant difference (*p* < 0.05), whereas similar letters in a row indicate no significant difference (*p* > 0.05). ^(1)^ DP, dressing percentage; SEWR, semi-eviscerated weight rate; ECWR, eviscerated carcass weight rate; PMR, pectoral muscle rate; LMR, leg muscle rate; AFR, abdominal fat rate. CON group, basal diet in the control group; GAA group, supplemented with 400 mg/kg guanidine acetic acid based on the control group diet; SEM, standard error of the mean.

**Table 4:** Effect of guanidine acetic acid on the meat quality of broiler chickens.

Items ^(1)^	BRT, L	MMR, %	SF, N	DL, %
CON	70.01	90.67	2.80 ^a^	4.48 ^a^
GAA	69.78	89.88	2.33 ^b^	4.34 ^b^
SEM	1.206	0.743	0.051	0.032
*p*-value	0.691	0.221	<0.001	<0.001

^a,b^ Means followed by different letters in a row indicate a significant difference (*p* < 0.05), whereas similar letters in a row indicate no significant difference (*p* > 0.05). ^(1)^ BRT, brightness; MMR, mature meat rate; SF, shear force; DL, drip loss. CON group, basal diet in the control group; GAA group, supplemented with 400 mg/kg guanidine acetic acid based on the control group diet; SEM, standard error of the mean.

**Table 5:** Effect of guanidine acetic acid on the serum biochemical indicators of broiler chickens.

Items ^(1)^	TP, g/L	TG, mmol/L	HDL, mmol/L	LDL, mmol/L	INS, mU/L	CGH, ng/ml	T3, nmol/L	T4, nmol/L
21 d								
CON	18.02	0.54 ^a^	2.16	1.28	22.57	8.82 ^b^	2.09 ^b^	189.04 ^b^
GAA	18.29	0.46 ^b^	2.24	1.31	23.17	10.04 ^a^	2.16 ^a^	197.45 ^a^
SEM	0.044	0.008	0.015	0.016	0.519	0.098	0.011	0.845
*p*-value	0.573	0.022	0.134	0.559	0.312	<0.001	0.024	0.005
42 d								
CON	18.45	0.53 ^a^	2.16 ^b^	1.09 ^b^	29.42	9.40 ^b^	4.39 ^b^	112.27 ^b^
GAA	18.48	0.49 ^b^	2.33 ^a^	1.33 ^a^	30.15	10.57 ^a^	5.51 ^a^	120.52 ^a^
SEM	0.050	0.004	0.017	0.023	0.429	0.093	0.088	0.647
*p*-value	0.431	0.002	<0.001	<0.001	0.557	0.013	<0.001	<0.001

^a,b^ Means followed by different letters in a row indicate a significant difference (*p* < 0.05), whereas similar letters in a row indicate no significant difference (*p* > 0.05). ^(1)^ TP, total protein; TG, triglyceride; HDL, high-density lipoprotein; LDL, low-density lipoprotein; INS, insulin; CGH, chicken growth hormone; T3, thyroid hormone T3; T4, thyroid hormone T4. CON group, basal diet in the control group; GAA group, supplemented with 400 mg/kg guanidine acetic acid based on the control group diet; SEM, standard error of the mean.

**Table 6:** Effect of guanidine acetic acid on the serum antioxidant capacity of broiler chickens.

Items ^(1)^	T-AOC, U/mL	SOD, U/mL	MDA, nmoL/mL	GSH-Px, U/mL
CON	0.14 ^b^	210.55 ^b^	10.21 ^a^	127.63
GAA	0.18 ^a^	261.91 ^a^	6.68 ^b^	129.66
SEM	0.002	12.187	0.339	7.163
*p*-value	<0.001	<0.001	<0.001	0.211

^a,b^ Means followed by different letters in a row indicate a significant difference (*p* < 0.05), whereas similar letters in a row indicate no significant difference (*p* > 0.05). ^(1)^ T-AOC, total antioxidant capacity; SOD, superoxide dismutase; MDA, malondialdehyde; GSH-Px, glutathione photooxygenase. CON group, basal diet in the control group; GAA group, supplemented with 400 mg/kg guanidine acetic acid based on the control group diet; SEM, standard error of the mean.

## Data Availability

The raw data supporting the conclusions of this article will be made available by the authors, without undue reservation. The raw data have been deposited in the NCBI Sequence Read Archive (SRA) database under the accession number PRJNA753623.
